# Outer membrane vesicles of *Acinetobacter baumannii* DS002 carry circular DNA similar to bovine meat and milk factors (BMMFs) and SPHINX 2.36 and probably play a role in interdomain lateral gene transfer

**DOI:** 10.1128/spectrum.00817-24

**Published:** 2024-08-05

**Authors:** Ganeshwari Dhurve, Sandhya Rani Behera, Gopinath Kodetham, Dayananda Siddavattam

**Affiliations:** 1 Department of Animal Biology, School of Life Sciences, University of Hyderabad, Hyderabad, Telangana, India; 2 Department of Plant Sciences, School of Life Sciences, University of Hyderabad, Hyderabad, Telangana, India; University of Florida, Gainesville, Florida, USA

**Keywords:** *A. baumannii*, outer membrane vesicles, OmpA, lateral gene transfer, bovine meat and milk factors

## Abstract

The discovery of Replication Competent Circular DNA molecules in mammalian cells and tissues is being linked to debilitating diseases, such as multiple sclerosis (MS), bovine spongiform encephalopathy (BSE), and colorectal cancer (CRC). These circular DNA molecules, otherwise known as bovine meat and milk factors (BMMFs) and Slow Progressive Hidden INfections of variable (X) latency (SPHINX), bear significant (80%) sequence similarity with the plasmids of *Acinetobacter baumannii* strains. Nanostructures, such as bacterial outer membrane vesicles (OMVs) serve as vehicles for transporting biomolecular cargo and have the potential to facilitate interkingdom lateral mobility of DNA. Strengthening the proposed hypothesis, this study demonstrates that OMVs derived from *A. baumannii* DS002 carrying four plasmids and genome (pTS236) of phage, AbDs1, successfully reached different parts of the body, including the central nervous system, following the injection of fluorescein isothiocyanate (FITC)-labeled OMVs into experimental mice. Out of the four OMV-associated plasmids, three (pTS4586, pTS9900, and pTS134338) were identified within the lumen, and the fourth one (pTS11291) was found on the surface of OMVs. In addition to the indigenous plasmids, the phage-encoded protein, Orf96, anchored on the surface of the OMVs by establishing a strong interaction with the OMV-associated porin, OmpA. Intriguingly, a subset of labeled OMVs, when incubated with Neuro2A cells, translocated across the membrane and reached to the cytoplasmic space of the cells. Collectively, the experimental evidence presented herein underscores the promising potential of OMVs as vehicles for delivering molecular cargo containing plasmids and phage genomes to diverse mammalian tissues and cells.

**IMPORTANCE:**

Several independent studies have demonstrated the existence of replication competent circular DNA molecules of bacterial and viral origin in mammalian cells and tissues. However, studies about their origin and lateral mobility to mammalian cells are scarce. Our work describes the existence of circular DNA, similar to that of DNA molecules identified in mammalian cells, OMVs derived from soil isolate of *A. baumannii* DS002. Furthermore, the work also provides visual evidence that demonstrates the passage of labeled OMVs to different organs of experimental mice within hours after intravenously administering OMVs into experimental mice. Some of the labeled OMVs have even crossed the membrane of Neuro2A, suggesting the existence of interkingdom horizontal mobility between bacteria and mammals.

## INTRODUCTION

Recent studies have unveiled the presence of replication-competent circular DNA molecules akin to plasmid and phage genomes within various mammalian tissues and body fluids (1–3). These circular DNA entities, termed as SPHINX DNAs, shielded by nucleases, have been notably observed in abundant numbers within highly infectious cytoplasmic particles extracted from Creutzfeldt–Jakob Disease (CJD) and scrapie specimens (4). Subsequent investigations have unearthed SPHINX like DNA sequences in both milk and meat samples obtained from healthy cattle as well as from the tissues of patients afflicted with conditions, such as MS and CRC. Alongside these SPHINX like sequences, various other replication competent circular DNA molecules have been identified within these tissues (3, 5). Dubbed as BMMFs, these circular DNA structures exhibit chimeric characteristics, integrating both plasmid and viral DNA sequences. Notably, they have been found in substantial concentrations within CRC tissue (6, 7). BMMFs have been linked to chronic inflammation, DNA damage, and are suggested to function as indirect carcinogens (7–9). Further corroborating BMMFs pathogenic potential, the presence of SPHINX sequences has been shown to modify the expression profile of human embryonic kidney cells (HEK293TT) (10).

The genus *Acinetobacter*, known for its strict aerobic nature, thrives across diverse ecological niches by metabolizing a wide array of toxic and recalcitrant organic compounds (11). Among these, certain strains of *A*.*baumannii* pose a significant threat as opportunistic pathogens, primarily targeting critically ill patients (12–14). Notably, desiccation-resistant variants of *A. baumannii* persist longer in hospital environments by adhering to surfaces of healthcare settings (15–17). Furthermore, analysis of plasmid and phage genomes from *A. baumannii* has unveiled sequence similarities with SPHINX and BMMF sequences (2). For instance, the genome of Phage AbDs1, isolated from *A. baumannii* DS002, exhibited a 67% similarity with SPHINX 2.36 and 80% similarity with one of the BMMF sequences (2, 18). Such notable sequence congruence across diverse sources hints at the prevalence of interdomain horizontal gene transfer (HGT).

*A. baumannii* strains also survive on the body surface of animals, and their presence in soil, water, and surfaces of hospital environments provides enormous scope for HGT between these two inseparable entities (19–21). OMVs of *A. baumannii* carry macromolecules, such as proteins and nucleic acids (22–30). The nanosized bacterial OMVs are resistant to enzyme degradation and low pH (31) and release adhesion molecules, facilitating adherence of bacteria to host tissues (32–34). OMVs released from bacteria follow different pathways to reach the bloodstream, and from there, they gain access to various tissues and possibly cross the blood–brain barrier (BBB) to reach the brain (35–37). In this study, we show the association of indigenous plasmids and phage AbDs1 genome with OMVs isolated from *A. baumannii* DS002 and demonstrate that the OMVs labeled with fluorescent dye FITC reach various organs of mice. Our findings suggest that the OMVs of *A. baumannii* DS002 serve as a potential source of the replication of competent-circular DNA identified in mammalian tissues.

## RESULTS

Several studies demonstrated instances of interdomain HGT involving bacteria and humans (19–21). The size of the human microbiome and its close association with somatic cells provides enormous scope for HGT between these two inseparable entities (20). OMVs secreted by bacteria carry macromolecules, such as proteins and DNA, and possess the remarkable capacity to traverse the BBB (35, 36, 38–42). A number of studies have convincingly established their role in the lateral mobility of bacterial DNA (43–45). In view of obvious sequence similarities between circular DNA molecules identified in mammalian tissues and *Acinetobacter* plasmids and phages, we have examined if OMVs isolated from *A. baumannii* DS002 have a role in delivering these extrachromosomal DNA molecules into mammalian cells.

### OMVs contain indigenous plasmids

**Fig 1:**
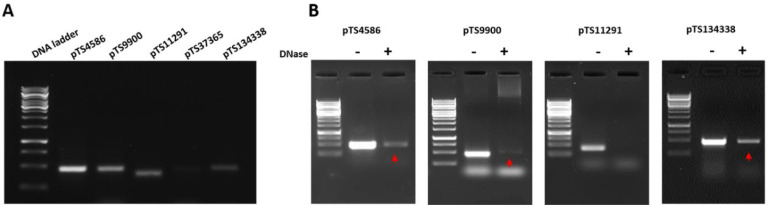
PCR amplification of plasmids associated with OMVs of *A. baumannii* DS002. A represents the amplification of DNA regions specific to indigenous plasmids of *A.baumannii* DS002. Lane 1 represents a kilobase ladder. Amplicons obtained when primers specific to pTS4586 (lane 2), pTS9900 (lane 3), pTS11291 (lane 4), pTS37365 (lane 5), and pTS134338 (lane 6) are shown in panel A. In lane 5 loaded with the reaction mix having primers specific to plasmid pTS37365, no amplification was observed, indicating the absence of plasmid pTS37365 in OMVs. B. Plasmid localization within OMVs: OMVs were treated with DNase to eliminate plasmid DNA present on the surface of the OMVs. The OMVs treated with DNase were used as template in the PCR reaction to detect plasmid present in the lumen. The amplicons obtained were then analyzed on 0.8% agarose gel along with the PCR amplicons obtained using DNase-untreated OMVs as a template. In all panels, lane 1 is the kilobase ladder, and lanes 2 and 3 represent PCR amplicons obtained when DNase untreated (−) and treated (+) OMVs were used as a template. The red arrow indicates amplicons obtained in DNase-treated OMVs. Indicating the presence of pTS11291 on the surface of OMVs, no amplification was noticed in the reaction mix with DNase-treated OMVs and primers specific to plasmid pTS11291. The amplicons specific to pTS4586, pTS9900, and pTS134338 were obtained, indicating their presence in the lumen of OMVs.

The pure OMVs isolated from *A. baumannii* DS002 cells were used for isolating total DNA (46). The procedure followed in the Methods section consistently yielded approximately 0.2 µg of DNA from 50 µg of OMVs. The isolated DNA was then sequenced, and the raw sequence reads were processed and assembled following standard protocols (11). These assembled sequences were then aligned with the genome sequence of strain DS002. Interestingly, all the assembled sequence contigs have matched only with four indigenous plasmids, pTS4586, pTS9900, pTS11291, and pTS134338 of *A. baumannii* DS002 (47). Notably, none of the contigs were aligned with the 37 kb plasmid pTS37365 identified in *A. baumannii* DS002. The sequence data were further validated by randomly amplifying the plasmid-specific sequences using pure OMVs as a template. In support of the sequence results, which indicated the selective exclusion of pTS37365 in OMVs, no pTS37365-specific sequences were amplified in PCR reactions (Fig. 1A). However, the amplicons specific to other four indigenous plasmids were obtained when PCR was performed using OMVs as template (Fig. 1A). The sizes of PCR amplicons coincided with the sizes of the corresponding regions of the indigenous plasmids pTS4586, pTS9900, pTS11291, and pTS134338 (Fig. 1A).

After ascertaining the presence of four indigenous plasmids, we designed further studies to establish their localization in OMVs. Initially, we have treated OMVs with DNase to eliminate any DNA associated with the surface of OMV. The DNase-treated OMVs were then used as templates in PCR reactions to amplify DNA corresponding to the four OMV-associated plasmids. Interestingly, we have obtained sequences specific to only plasmids pTS4586, pTS9900, and pTS134338 (Fig. 1B). There was no amplification in the reaction mix that contained pTS11291-specific primers, suggesting that the DNase treatment eliminated plasmid pTS11291 (Fig. 1B, panel III). DNase digestion failed to eliminate plasmids pTS4586, pTS9900, and pTS134338 as the membrane of OMVs prevented gaining access to these plasmids (Fig. 1B). We have performed these studies on two biological replicates, and in both cases, we have obtained identical results.

### Phage AbDs1 is associated with OMVs

**Fig 2:**
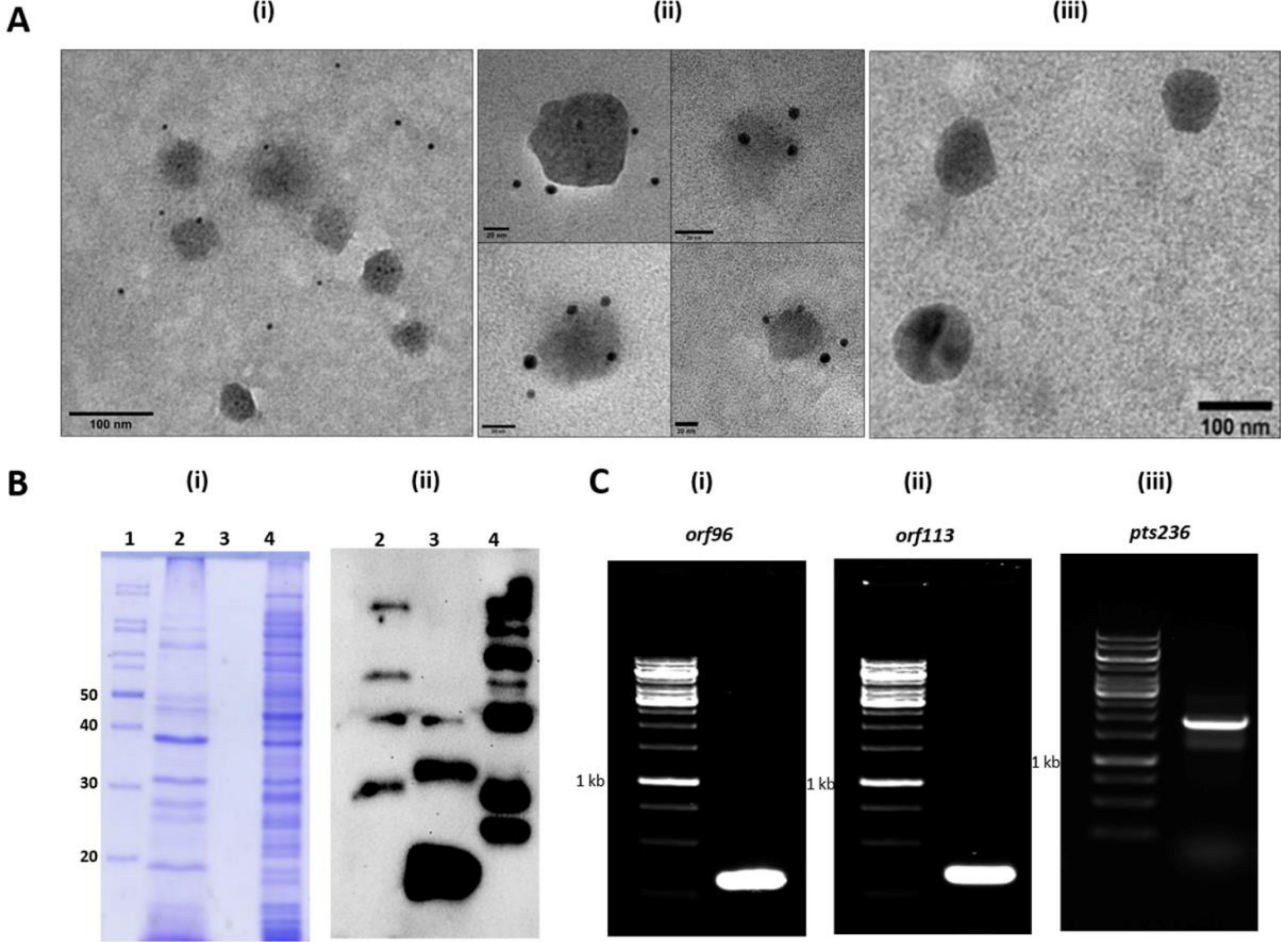
Detection of phage AbDs1-specific Orf96 in OMVs. A (i) indicates a broad field used to take image showing the presence of gold particles around OMVs, A (ii) represents independent OMV showing the presence of gold particles around them. A (iii) shows TEM images of OMVs after treatment with only anti-Orf96 antibody. B (i) shows 12% SDS-PAGE. Lane 1 represents protein ladder, lane 2 is OMV proteome, lane 3 contains pure Orf96^C6XHis^ protein and 4 shows total lysate of *A. baumannii* DS002. B (ii) corresponds to Western blot probed with polyclonal antibody specific to Orf96. C image indicates amplification of the phage genome using primers specific to different ORFs of phage AbDs1 genome (pTS236). C (i), (ii), and (iii) represent amplicons obtained for *orf96*, *orf113*, and complete genome (pTS236) of phage AbDs1 by using OMVs as templates, respectively.

The experimental conditions used for the analysis of OMV DNA sequences did not reveal the presence of sequences specific to phage AbDs1 DNA (pTS236). The minimum cut off length (4 kb) of contigs used for aligning with the genome sequence of *A. baumannii* DS002 naturally eliminates inclusion of 2.36 kb phage genome from the sequence analysis. Our previous proteomic studies conducted to identify OMV-associated proteins (Proteome Exchange via PRIDE with the identifier PXD026751) revealed the presence of phage AbDs1-specific protein, when searched against pTS236 proteome (data not shown). This preliminary lead was verified by obtaining TEM images as well as Western blots. Initially, we treated the OMVs with Orf96-specific antibodies available in our laboratory (18) and obtained TEM images after incubating them with gold-labeled secondary antibodies. The TEM images showed the presence of gold-labeled secondary antibodies on the surface of OMVs (Fig. 2A), indicating the existence of phage AbDs1-specific protein, Orf96 on the surface of OMVs (Fig. 2Ai and ii ). Supporting the TEM images, the Western blots also revealed the presence of Orf96 in OMVs. However, the Orf96-specific signals obtained in the lanes loaded with total OMV proteins (Fig. 2B lane 2), affinity-purified Orf96 (Fig. 2B lane 3), and total soluble proteins of *A. baumannii* DS002 appeared in the form of a ladder (Fig. 2B lane 4), suggesting the formation of SDS resistant Orf96 multimers. Multimerization of SDS resistant protein is quite common, especially in virus-coded proteins (48). Since a similar trend is seen with respect of Orf96, we assume that Orf96 is a coat protein of AbDs1. After establishing the presence of phage AbDs1-specific Orf96 in OMVs, we have done PCR using pure OMVs as template to amplify ORFs identified in AbDs1 genome pTS236 (18). Intriguingly, amplicons corresponding to the size of two ORFs, *orf96* (panel C-i), *orf113* (panel C-ii) and the complete genome of AbDs1 (panel C-iii) were obtained, suggesting the presence of a complete genome of AbDs1 in OMVs.

### Orf96 anchors to OMVs by interacting with OmpA

**Fig 3:**
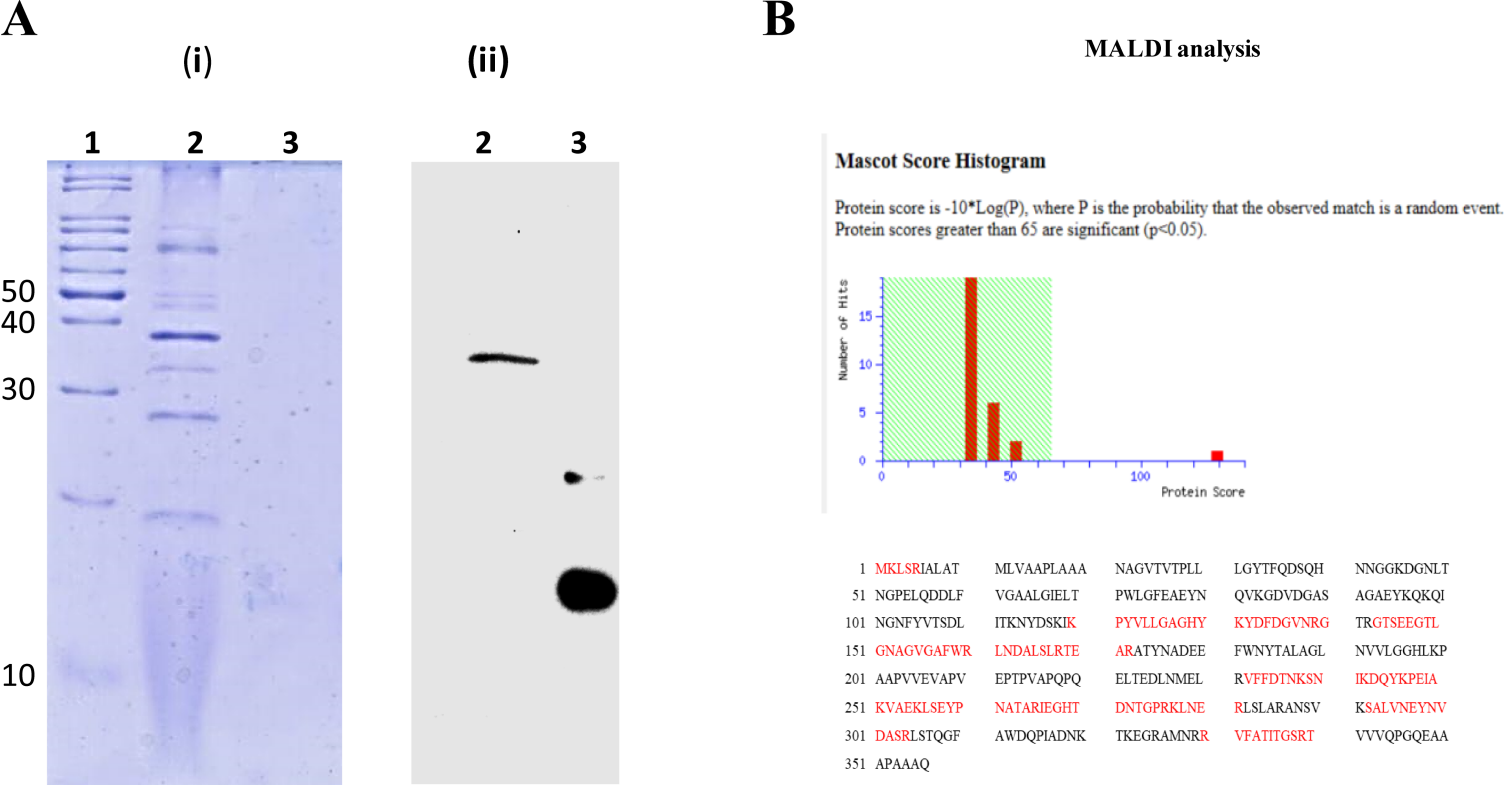
Ligand blot assay for detection of OMV proteins interacting with phage genome-coded Orf96. A (i) shows 12.5% SDS-PAGE stained with Coomassie blue. Lane 1 represents protein ladder, lane 2 shows OMV protein, and lane 3 represents pure Orf96^C6XHis^ used as positive controls. Unstained reference gel was used to transfer proteins onto the polyvinylidene difluoride (PVDF) membrane, and the blot was incubated with affinity-purified Orf96^C6XHis^ . The blot was then probed with anti-His antibodies. A (ii) shows a clear signal detected below the size of 40 kDa marker protein in lane loaded with OMV proteins (lane 2), and the signal obtained with pure Orf96^C6XHis^ (11 kDa) loaded as positive control is shown in lane 3. Mascot ID generated for 37 kDa protein band interacting with Orf96^C6XHis^ has matched with OmpA and is shown in B. The sequences highlighted in red indicate the identity between the generated peptide sequences through MALDI TOF/TOF and the sequence of OmpA predicted from the genome sequence of *A. baumannii* ATCC 19606.

After establishing the presence of AbDs1-specific protein, Orf96, and its genome pTS236, further experiments were designed to elucidate the mechanism of anchoring of AbDs1-specific Orf96 with OMVs. Assuming that Orf96 is interacting with OMV-specific protein, we performed ligand blots to identify Orf96 interacting partner among OMV proteins. The affinity-purified Orf96^C6XHis^ strongly interacted with a OMV protein of 37 kDa and contributed for a clear signal when the blot was probed with anti-His antibody (Fig. 3A). The peptide finger print pattern and the sequences of randomly obtained peptides of the 37 kDa protein band perfectly matched with outer membrane porin, OmpA (Fig. 3B).

**Fig 4:**
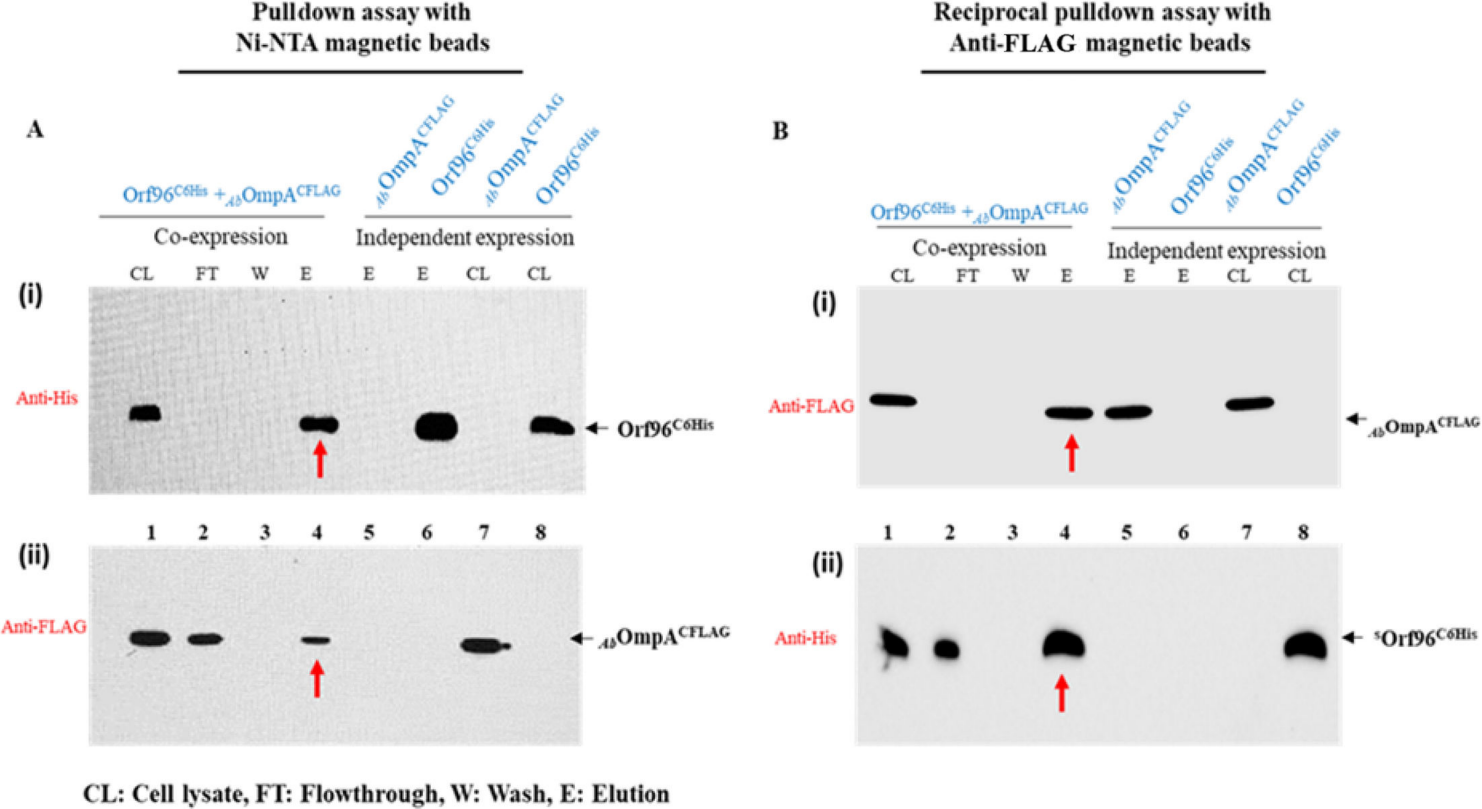
Pulldown assays were performed to demonstrate interactions between Orf96 and OmpA. Pulldown assays performed using Ni-NTA and FLAG magnetic beads are shown in A and B, respectively. The cell lysates prepared from cells (pGD3+pT96W) co-expressing OmpA^CFLAG^ and Orf96^C6His^ and separately (independent expression) were used to perform pulldown assays. CL represents cell lysate used as input. The FT, W, and E indicate flowthrough, wash, and elution fractions, respectively. A (i) indicates Western blots performed using anti-His antibodies. Similar experiments done using anti-FLAG antibodies are shown in A (ii). Lane CL contains both OmpA^CFLAG^ and Orf96^C6His^ specific signals. A significant amount of OmpA^CFLAG^ is seen in lane FT. No signals were seen in lane W loaded with wash due to dilution of wash fraction. The OmpA^CFLAG^ co-eluted along with Orf96^C6His^ is shown with a red arrow. The reciprocal pulldown shown in B indicates a similar loading and blotting pattern. The Orf96^C6His^ co-eluted with OmpA^CFLAG^ is shown with the red arrow in B (ii).

These results were further confirmed by performing reciprocal pulldowns by co-expressing OmpA and Orf96 with different affinity tags. The OmpA was expressed with a C-terminal FLAG tag, whereas Orf96 contained C-terminal His-tag. The *Escherichia coli* cell lysate containing these two proteins was used to perform pulldown assays using either nickel magnetic beads or magnetic beads tagged to anti-FLAG antibodies. The proteins purified using magnetic beads were then analyzed on SDS-PAGE, and Western blots were performed by using both anti-FLAG and anti-His antibodies to detect OmpA^CFLAG^ and Orf96^C6XHis^. When nickel magnetic beads were used to pulldown Orf96^C6XHis^, we have always seen co-elution of OmpA^CFLAG^ (Fig. 4A). In similar pulldown experiments performed using magnetic-FLAG tag antibodies along with OmpA^CFLAG^, we have seen co-elution of Orf96^C6XHis^ (Fig. 4B). These reciprocal pulldown assays clearly indicated interactions of phage AbDs1 protein Orf96 with OmpA.

### OMVs cross the blood–brain barrier

**Fig 5:**
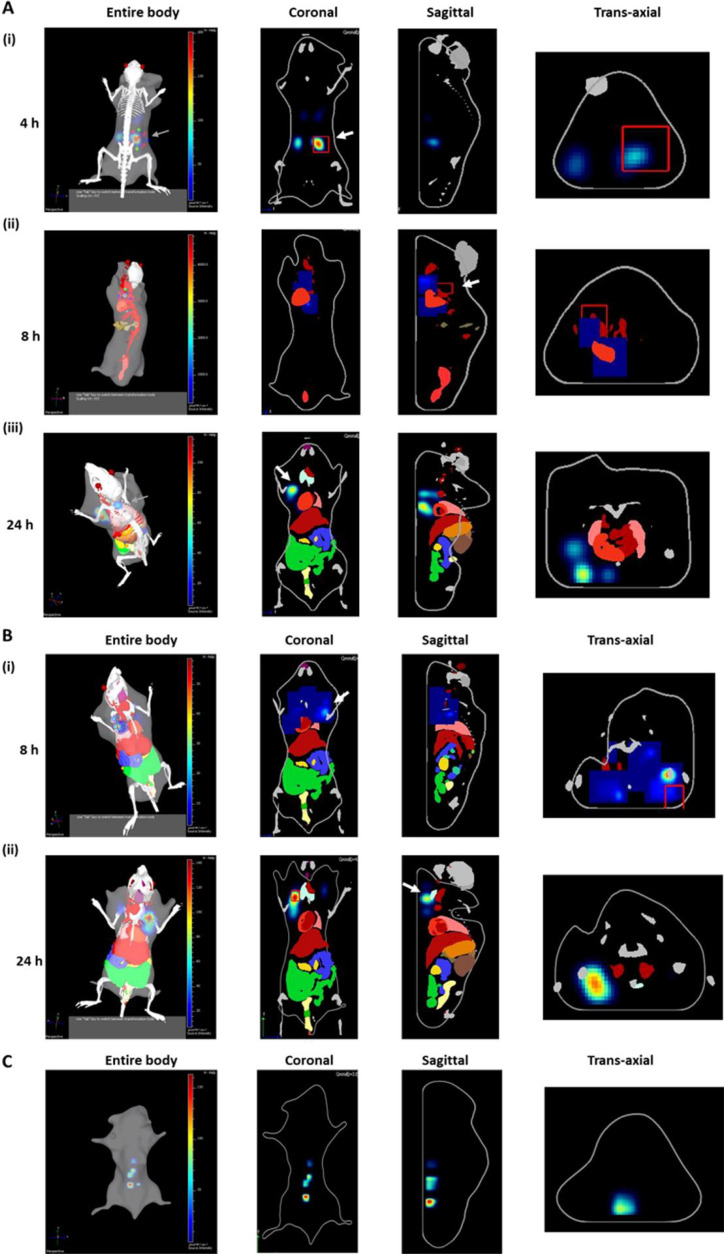
Female BALB/c mice were intravenously administered with various concentrations of FITC-labeled outer membrane vesicles (OMVs), and the *in vivo* images were obtained in different anatomical planes, including coronal, sagittal, and trans-axial using *in vivo* imaging system (Perkin-Elmer IVIS Spectrum). A and B show images obtained for mice administered with 5 µg (A) and 20 µg (B) of FITC-labeled OMVs, respectively. Images obtained for mice after 4 h (A (i)) and 8 h (A (ii)) post-injection display the accumulation of OMVs in the kidney and circulatory system (highlighted by an arrow). In 24 h (A (iii)), the OMVs were detected in the spinal cord. B depicts mice injected with 20 µg of OMVs in 8 h post-injection, and the presence of fluorescence indicates OMV presence in the lymphoid system (B (i)) and in 24 h post-injection, some of them reached proximal to the brain (B (ii)). C represents images of the control group receiving sterile PBS, and fluorescence images obtained 24 h post-injection served as a baseline. These observations reveal distinct and time-dependent distribution patterns of systemically administered OMVs across various anatomical regions, including the kidney, circulatory system, spinal cord, lymphoid system, and brain vicinity.

**Fig 6:**
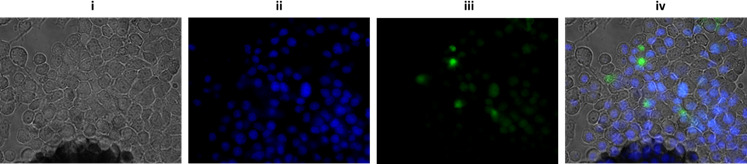
Confocal microscopy pictures of Neuro2A cells taken at different fluorescence channels. Images were taken after incubation of the Neuro2A cells with FITC-labeled OMVs showing translocation of OMVs into Neuro2A cells. i (black and white channels) shows neuronal cells. ii shows nucleus stained with DAPI, and iii is OMVs labeled with FITC. iv is the merged image, indicating the presence of OMVs inside some of the Neuro2A cells.

Several independent studies have suggested OMVs crossing the BBB by using both paracellular and transcellular pathways (35–37). The OMV-associated proteins and enzymes modulate the permeability of cellular junctional complexes by cleaving the proteins associated with adherent junctions and tight junctions (39, 49). In light of these observations, we have conducted further experiments to examine if the OMVs of *A. baumannii* DS002 have any role in transporting the genome of phage AbDs1 and associating plasmids to various mammalian cells and tissues. In our initial experiments, we labeled OMV proteins with fluorescent dye FITC, and the labeled OMVs were intravenously injected into mice at the dosage of 5 µg, 20 µg and 30 µg. Mice injected with 30 µg of OMVs failed to survive. Therefore, the *in vivo* whole-body live images were obtained only for the animals injected with 5 µg and 20 µg OMVs, respectively. As seen in the images, the injected OMVs reached to various parts of the body, including the central nervous system (Fig. 5, panel A (ii) and panel B (ii)). They reached to the kidneys within 4 h (Fig. 5 panel A (i)) and were found in the circulatory system after 8 h (Fig. 5, panel A (ii)). The images obtained after 24 h have shown the presence of OMVs in spinal cords and even reached to the proximity of the brain in animals injected with 20 µg of OMVs (Fig. 5, panel B (ii)). After noticing the presence of OMVs in different organs of mice, we performed additional experiments to determine if they could be internalized into mammalian cells. The FITC-labeled OMVs were incubated with Neuro2A cell lines and visualized under the confocal microscope. Some of the OMVs successfully crossed the membrane of Neuro2A cells and emitted fluorescence light, indicating the internalization of OMVs within the Neuro2A cells (Fig. 6). Presumably, the translocated OMVs delivered associated plasmids and phage AbDs1 genome into animal cells.

## DISCUSSION

Several independent studies have reported the presence of circular chimeric DNA in mammalian cells and tissues (50). Out of them, SPHINX DNA sequences identified in the brain samples of animals suffering from BSE and BMMFs, isolated from cattle milk and meat, acquire a lot of significance. The BMMF and SPHINX DNA sequences were selectively enriched in tissues of patients suffering from dreadful diseases, such as CRC, MS, and BSE (1, 2, 51). The BMMFs isolated to date are categorized into four distinct phylogenetic groups, such as BMMF-I, BMMF-II, BMMF-III, and BMMF-IV (2, 5). Of these four groups, three (BMMF-I to BMMF-III) share sequence similarities with the indigenous plasmids of *A. baumannii*. The fourth group (BMMF-IV) contains sequences similar to genome sequence of Gemycircularvirus (2). All of them have at least one ORF that codes for replication function and is highly conserved among all BMMFs and SPHINX sequences (52, 53). Interestingly, in human HEK293TT cells, the *rep* gene is expressed using transcriptional and translational machinery of host cells and supports its stable maintenance (10). In addition to the Rep protein, mass spectrometry analysis has shown the presence of BMMF-coded peptides in HEK293TT cells (10). Expression of these BMMF-coded peptides impacted the expression profile of host cells, especially those controlling cell cycle and cell viability (10). Although the direct link between the expression of BMMF-coded proteins and induction of diseases, such as MS, BSE, and CRC is lacking, the existence of antibodies in animal sera against the BMMF-coded proteins indirectly indicates their potential role in the induction of these diseases (2, 7).

OMVs play a key role in carrying the molecular cargo containing DNA and proteins from bacteria to mammalian cells and tissues (23, 54, 55). OMVs, which appear as spherical buds ranging from 20 to 400 nm contain enzymes, nucleic acids, metabolites, and toxins (32). The lipid bilayer of the OMV protects the biomolecular cargo from the harsh extracellular environments and safely transports them to distant places (31, 55). The OMVs released by pathogenic and commensal bacteria reach either gastrointestinal (GI) tract or directly to the blood stream. The OMVs released into GI tract cross the intestinal epithelium and vascular endothelium by following either paracellular or transcellular pathways to finally reach the blood stream (31). After reaching the blood stream, the OMVs travel to various organs and tissues and even cross the BBB to deliver macromolecules, such as DNA and other virulence factors, to various tissues, including the brain (37, 56–58).

Our previous studies have identified circular DNA molecule, pTS236, while analyzing plasmids of *A. baumannii* DS002 (18). The circular DNA molecule, pTS236, later identified as genome of phage, AbDs1, showed a significant sequence similarity with SPHINX 2.36 and class-II BMMFs, isolated from bovine meat and milk samples (2). Such high sequence similarities between DNA molecules isolated from taxonomically diverse sources suggest the existence of interdomain lateral gene transfer event. *A. baumannii* strains survive in a variety of ecological niches, including on the body surface of animals and plants. Some of them are opportunistic pathogens and contribute significantly to hospital-acquired infections (59). There is ample scope for the OMVs released by the free-living *A. baumannii* strains to reach the GI tract through the food chain and finally to the blood stream. Likewise, the pathogenic strains of *A. baumannii* directly deliver their OMVs into the blood stream. Once they reach the circulatory system, the OMVs successfully cross the BBB to reach the brain (35, 37). Corroborating with these studies, the labeled OMVs administered intravenously into mice have reached different organs and tissues of the mice (Fig. 5), and some of them even crossed the membrane of the cells to reach the cytoplasmic space (Fig. 6). Such a voyage of OMVs delivers associated DNA into the cells and also exposes its genome to the transcriptional and translational machinery of host cells. Some of the genes of OMV-associated DNA, particularly the *rep* gene, are expressed in eukaryotic cells (10). The expressed Rep protein has even contributed to the replication of circular DNA molecules in mammalian cells (10). These reports, if seen together with the conservation of *rep* genes among BMMFs and SPHINX, very well explain the molecular basis behind the existence of a large number of circular DNA molecules in mammalian cells. The data reported in this study provide conclusive evidence on the origin of circular DNA found in mammalian tissues and cells and highlight the role of OMVs in lateral mobility of bacterial DNA to mammalian cells.

## MATERIALS AND METHODS

### Bacterial strains and plasmids

Bacterial strains and plasmids used in this study are listed in supplementary table (Table S1). The *E. coli* and *A. baumannii* DS002 cultures were grown in LB medium at 37°C and 30°C, respectively. When necessary, antibiotics chloramphenicol (30 µg/mL) and streptomycin (20 µg/mL) were supplemented to the culture medium.

### Isolation of total DNA from OMVs

The OMVs of *A. baumannii* DS002 were isolated following established procedures described elsewhere (46). The purified OMVs (50 µg of protein concentration) were initially adjusted to 2% Triton X-100 concentration by adding the necessary volume of a stock solution. This mixture was then thoroughly blended and incubated at 55°C for 10 min to solubilize the membrane of OMVs. The Triton X-100 treated OMVs were then used to isolate total DNA by using QIAGEN Genomic-tip Kit following the manufacturer’s protocols. Briefly, the lysate of OMVs was mixed with an equal volume of binding buffer and loaded onto the QIAGEN genomic DNA extraction column. Subsequently, the column was washed twice with a wash buffer and the DNA bound to the column was eluted with 20 µL of sterile water. While isolating DNA present inside the lumen, the OMVs were incubated with 2 Units of DNase for 30 min at 37°C to eliminate surface-associated DNA. Subsequently, the DNase was inactivated by incubating OMVs at 80°C for 10 min. Following DNase treatment, the OMVs were used to isolate DNA present within the lumen of OMVs, following the procedures described above.

### Detection of plasmids and phage AbDs1 in OMVs

Our previous studies have reported the complete genome sequence of *A. baumannii* DS002. In addition to the 3,430,798 bp long chromosome, *A. baumannii* DS002 contains five indigenous plasmids and phage AbDs1 genome (11). The DNase treated and untreated OMVs were used as a source of template, and PCR was performed using primers specific to indigenous plasmids and phage AbDs1 DNA (Table S2). If plasmid-specific amplicons were obtained both in DNase treated and untreated OMVs, they were considered as present in the lumen. If the amplicons were seen only in DNase-untreated OMVs they were assumed as present on the surface of the OMV.

### Detection of phage AbDs1-specific proteins in OMVs

The presence of phage AbDs1 genome-coded proteins in OMVs of *A. baumannii* was determined by performing both Western blots and immunogold labeling techniques. Initially, OMV proteins equivalent to 30 µg were separated on 12% SDS gel, and Western blots were performed using antibodies specific to one of the phage AbDs1 genome-coded protein, Orf96 (18). Because Western blots indicated the presence of phage AbDs1-coded protein, Orf96 in OMVs, TEM images were obtained to establish its precise location in OMVs. Initially, the OMVs were treated with Orf96-specific primary and gold-labeled secondary antibodies, and TEM images of OMVs were taken following procedures described elsewhere (46). Briefly, different concentrations of purified OMVs were spotted onto copper grids and rinsed them by floating on a droplet of distilled water before incubating in a blocking buffer (0.3% BSA in PBS) for 15 to 30 min. Then, the grids were thoroughly washed with a wash buffer (0.03% BSA in PBS) and were incubated with anti-Orf96 antibodies for 2 h. Subsequently, the grids were washed extensively to remove excess antibodies and carefully submerged in a droplet of buffer containing gold-conjugated secondary antibodies for 1 h. After incubation, the grids were washed three times each with the wash buffer and water. Excess liquid was removed by gently touching the grids with filter paper. Finally, the grids were stained with 2% uranyl acetate for 1 min before obtaining images using a transmission electron microscope (TEM).

### Ligand blotting

The OMV-associated protein interacting with phage-encoded Orf96 was identified by performing ligand blot experiment. OMV proteins separated on 12% SDS-PAGE were transferred onto a polyvinylidene fluoride (PVDF) membrane and blocked with 3% BSA in TBST (Tris-Buffered Saline with Tween). The blocked membrane was then incubated at room temperature for 1 h in TBST containing 0.2% BSA and affinity-purified Orf96^C6His^ protein (1 µg/mL). The membrane was then extensively washed with TBST, and the presence of Orf96^C6His^ was detected by using anti-His-HRP-conjugated antibody. The protein band that gave signal was carefully excised from the reference gel, and its identity was established by performing Matrix-Assisted Laser Desorption Ionization-Time of flight (MALDI-TOF/TOF ) (60).

### Reciprocal pulldown assays

Reciprocal pulldown assays were performed to ascertain interactions between OmpA and Orf96. Initially, two compatible expression plasmids were constructed to express Orf96 and OmpA with C-terminal 6XHis and FLAG sequences, respectively. The *ompA* gene was amplified from genomic DNA as *NdeI* and *XhoI* fragments using the primer set GD2FP/GD2RP (Table S2) and ligated in pET23b. The resulting plasmid designated as pGD2 was then used as a template and T7FP/T7RP as primers to amplify *ompA* gene as a *BglII* fragment (Table S2). The amplicon was then cloned into a broad host range mobilizable expression plasmid pRGOOD digested with *BamHI*. In the resulting recombinant plasmid, pGD3, the *ompA* gene is under the transcriptional control of the pBAD promoter and codes OmpA^CFLAG^. The *E. coli* BL21 DE3 (pGD3) cells were then transformed with a second expression plasmid, pT96W, which codes Orf96^C6His^(18) to coexpress OmpA^CFLAG^. The *E. coli* BL21 DE3 (pGD3+pT96W) cells were grown to mid-log phase, and the expression of both Orf96^C6His^ and OmpA^CFLAG^ was induced by adding 1 mM isopropyl β-D-1-thiogalactopyranoside (IPTG) and 1% arabinose. Expression of both Orf96^C6His^ and OmpA^CFLAG^ was detected by performing Western blots using either anti-His or anti-FLAG antibodies, and the clear lysate prepared from the induced cells was used to perform reciprocal pulldown assays using either Ni-NTA or anti-FLAG M2 magnetic beads following protocols optimized in our laboratory (61).

### FITC labeling of OMVs

OMVs isolated from *A. baumannii* DS002 were labeled using FITC following the procedure described elsewhere (62). Briefly, the isolated OMVs (1 mg/mL) were diluted and mixed in 1:1 ratio with FITC stock solution prepared by dissolving in buffer containing 50 mM sodium carbonate and 100 mM sodium chloride (pH 9.2). The mixture was then incubated for 1 h at 25°C before pelleting the labeled OMVs by centrifuging the contents at 150,000×*g* for 90 min. The OMVs were then washed with a PBS to remove excess FITC, and the labeled OMVs were pelleted by repeating the centrifugation process. The resulting OMV pellet was resuspended in PBS and filtered through a 0.22 micron filter before examining the labeled OMVs under fluorescence spectroscopy.

### *In vivo* imaging

The experimental mice were treated in accordance with the guidelines established by the Institutional Animal Ethics Committee (IAEC) (Proposal number:UH/IAEC/SD/2021-1/46) of School of Life Sciences, University of Hyderabad, India. Female BALB/c mice of the same age and weight were acclimatized to laboratory conditions before using them to track the mobility of intravenously injected FITC-labeled OMVs. Initially, the mice were divided into four groups, each consisting of 6-weeks-old two female BALB/c mice. One of them served as control group, and the mice of the control group were administered 200 µL solution of PBS instead of FITC-labeled OMVs. The FITC-labeled OMVs at concentrations of 5 µg, 20 µg, and 30 µg were drawn into a clean syringe and injected intravenously into the mice of three experimental groups, respectively. The mice were then sedated to minimize the movement and whole-body imaging was done at the time intervals of 4, 8, and 24 h by using an *in vivo* imaging system (Perkin-Elmer IVIS Spectrum). Analysis of captured images was done following procedures described elsewhere (63). Images of sedated mice were recorded using 480/520 nm excitation and emission filters to facilitate visualization of fluorescence signals within the chosen area. Initially, the acquired images and specific regions of interest (ROIs) were delineated, and the fluorescence within these ROIs was quantified using the standardized unit "Radiant Efficiency (p/s/sr)/(µW/cm2)".

### OMV internalization assay

Neuronal cells (Neuro2A) cultured in Dulbecco’s Modified Eagle’s Medium (DMEM) with 10% FBS were seeded on coverslips and incubated until reaching 70%–80% confluency. The media were replaced with fresh DMEM, and 20 µg of FITC-labeled OMVs was added for a 12-h incubation. Following incubation, cells were carefully washed with PBS, fixed using 4% paraformaldehyde, and mounted on slides. Finally, fluorescence microscopy was used to visualize and assess the internalization of FITC-labeled OMVs within the Neuro2A cells.

## SUPPLEMENTAL MATERIAL

Supplementary material spectrum.00817-24-s0001.docx Tables S1 and S2.

ASM does not own the copyrights to Supplemental Material that may be linked to, or accessed through, an article. The authors have granted ASM a non-exclusive, world-wide license to publish the Supplemental Material files. Please contact the corresponding author directly for reuse.
