# Supplementary material for: Outer membrane vesicles of Acinetobacter baumannii DS002 carry circular DNA similar to bovine meat and milk factors (BMMFs) and SPHINX 2.36 and probably play a role in interdomain lateral gene transfer
Source: Microbiol Spectr. 2024 Aug 5;12(9):e00817-24. doi: 10.1128/spectrum.00817-24 (PMC11370262; doi:10.1128/spectrum.00817-24)
Supplement: Supplementary material — Tables S1 and S2. [file spectrum.00817-24-s0001.docx]

| **Strains** | **Source and description** | **References** |
| --- | --- | --- |
| *E. coli* DH5α | *λsupE44, ΔlacU169 (Δ80 lacZΔM15) hsdR17 recA1 endA1 gyrA96 thi1 relA1* | (1) |
| *E. coli* BL21 | *hsdS* *gal* (λclts857 ind1 sam7 nin5lac uv5 T7 gene 1) | (2) |
| *Acinetobacter baumannii* DS002 | Cm^r^, Sm^r^ | (3) |
| **Plasmids** | |  |
| pET23b | Amp^r^, expression vector, codes proteins of cloned genes with C‑terminal His-tag. | Novagen,USA |
| pT96W | Amp^r^, Generated by ligating *orf96* of pTS236 as *EcoRI* and *XhoI* fragment in pET23b. Codes for Orf96^C6His^. | (4) |
| pGD2 | Amp^r^, Expression plasmid. Generated by ligating *ompA* in pET23b as *Nde*I and *Hind*III. Codes for OmpA^CFLAG^. | This Study |
| pGD3 | Cm^r^, Expression plasmid. Generated by ligating *ompA* in pRGOOD vector as *Bgl*II fragment. Codes OmpA^CFLAG^ from an arabinose inducible PBAD promoter. | This study |

**Supplementary Table 1: Bacterial strains and plasmids**

**Supplementary Table 2: primers**

| **Name** | **Sequence** | **Purpose** |
| --- | --- | --- |
| GD2FP | TACTCG*CATATG*AAATTGAGTCGTATTGCACTTGCTACTA | Forward primer used to amplify *ompA* gene of *A. baumannii* DS002. The *Nde*I site appended to facilitate ligation of the amplicon in pET23b is underlined. |
| GD2RP | ATCA*AAGCTT*TTA**CTTGTCGTCATCGTCTTTGTAGTC**TTGAGCTGCTGCAGGAGCTG | Reverse primer used to amplify *ompA* gene of *A. baumannii* DS002.The sequence specifying FLAG tag introduced by replacing the stop codon is shown with bold case. The *Hind*III site appended down stream of stop codon to facilitate ligation of the amplicon in pET23b is underlined. |
| GD3 FP | CCTT*AGATCT*TAATACGACTCACTATAGG | Forward and reverse primers specific to pET23b vector, used to amplify variant of *ompA* from pGD2 as *Bgl*II fragmnet. The *Bgl*II site appended to facilitate ligation of amplicon in pRGOOD is underlined. |
| GD3 RP | CCTT*AGATCT*CTAGTTATTGCTCAGCGGTGGC |  |
| GD4 FP | GCATTGATGGCAGTTGCAGAAC | Primers used to amplify plasmid pTS4586 specific sequence. |
| GD4 RP | GGGTCTATGACACAAAAATCGCC |  |
| GD5 FP | CTAATTCACGCTTTCCGCCAAAAACC | Primers used to amplify plasmid pTS11291 specific sequence. |
| GD5 RP | CAGAGATAAATACAGCTTTAGTGTGTTCTG |  |
| GD6 FP | CGATTTCTACCAGCTGGTATTGC | Primers used to amplify plasmid pTS9900 specific sequence. |
| GD6 RP | GATAATTAGTGCTATCCACTTTAACCC |  |
| GD7 RP A | GTCATGCCACAAGCTCAAGCAGG | Primers used to amplify plasmid pTS37365 specific sequence. |
| GD7 RP A | GTCTGCATTGATGGACCTACG |  |
| GD7 RP B | CCCTGTAGAAGATCGTGATCCTGC | Primers used to amplify plasmid pTS37365 specific sequence. |
| GD7 RP B | GCTAACAAATGGTTGAAGCTCAACAAACC |  |
| GD8 RP A | CGTCTGCCTGAATTTGATGAATGGCTAG | Primers used to amplify plasmid pTS134338 specific sequence. |
| GD8 RP A | CTCGTCCAATACCTGCTTCTTTCGC |  |
| GD8 RP B | GGCTGGAAATGCATATGCTGAC | Primers used to amplify plasmid pTS134338 specific sequence. |
| GD8RP B | GGTGAGCTGACGAGAATCATC |  |
| GD8 FP C | CGACATAATGGAGTAGTCAGCACTAG | Primers used to amplify plasmid pTS134338 specific sequence. |
| GD8 RP C | CATCTTCCCAACGGAGTTCACG |  |

**References**

1. Hanahan D. 1983. Studies on transformation of Escherichia coli with plasmids. J Mol Biol 166:557-80.

2. Studier FW, Moffatt BA. 1986. Use of bacteriophage T7 RNA polymerase to direct selective high-level expression of cloned genes. J Mol Biol 189:113-30.

3. Yakkala H, Samantarrai D, Gribskov M, Siddavattam D. 2019. Comparative genome analysis reveals niche-specific genome expansion in Acinetobacter baumannii strains. PLoS One 14:e0218204.

4. Longkumer T, Kamireddy S, Muthyala VR, Akbarpasha S, Pitchika GK, Kodetham G, Ayaluru M, Siddavattam D. 2013. Acinetobacter phage genome is similar to Sphinx 2.36, the circular DNA copurified with TSE infected particles. Sci Rep 3:2240.
